# Potential Role of Vitamin B6 in Ameliorating the Severity of COVID-19 and Its Complications

**DOI:** 10.3389/fnut.2020.562051

**Published:** 2020-10-29

**Authors:** Thanutchaporn Kumrungsee, Peipei Zhang, Maesaya Chartkul, Noriyuki Yanaka, Norihisa Kato

**Affiliations:** ^1^Graduate School of Integrated Sciences for Life, Hiroshima University, Higashi-Hiroshima, Japan; ^2^State Key Laboratory of Cellular Stress Biology, School of Medicine and School of Life Science, Xiamen University, Xiamen, China; ^3^Emergency Department, Bangkok Chanthaburi Hospital, Chanthaburi, Thailand; ^4^Emergency Department, King Prajadhipok Memorial Hospital, Chanthaburi, Thailand; ^5^Department of Preventive and Social Medicine, Faculty of Medicine, Siriraj Hospital, Mahidol University, Bangkok, Thailand

**Keywords:** vitamin B6, COVID-19, pneumonia, cardiovascular diseases, diabetes, inflammasome, oxidative stress, carnosine

## Introduction

The word is currently experiencing a coronavirus disease-19 (COVID-19) pandemic caused by a novel coronavirus, severe acute respiratory syndrome coronavirus 2 (SARS-CoV2). Coronaviruses and influenza are among the viruses that can cause lethal lung injuries and death from acute respiratory distress syndrome worldwide (1). Viral infections evoke a “cytokine storm,” leading to lung capillary endothelial cell inflammation, neutrophil infiltration, and increased oxidative stress (1, 2). Furthermore, cardiovascular and diabetic complications are emerging in COVID-19 patients (3–9). Currently, there is no registered treatment or vaccine for COVID-19, and an alternative solution to protect against COVID-19 is urgently needed.

**Figure 1:**
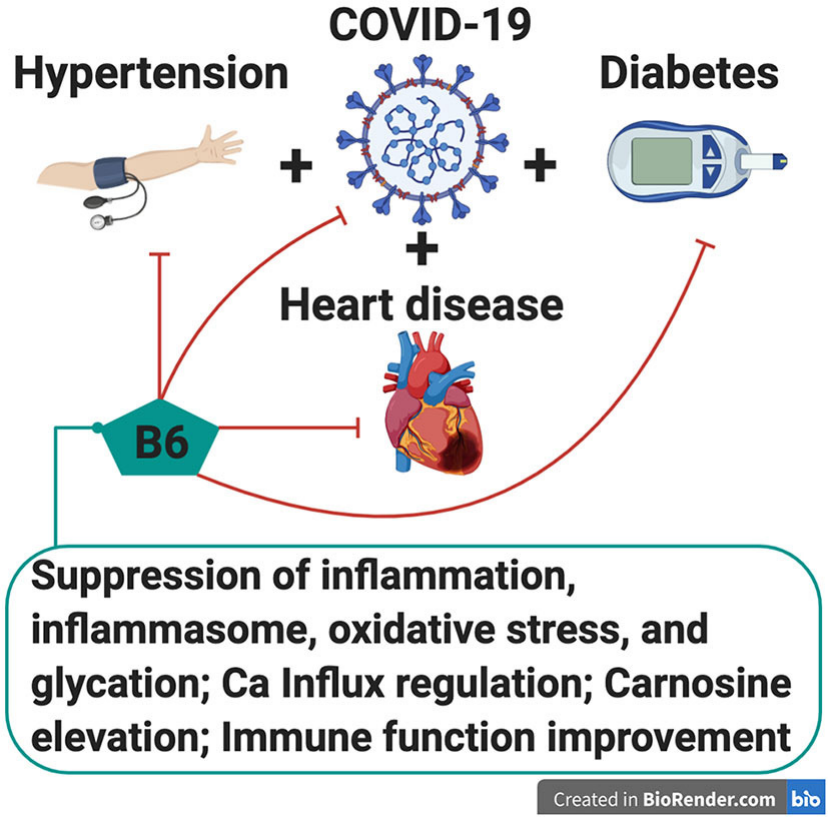
Potential protective role of vitamin B6 in ameliorating the severity of COVID-19 and its complications such as hypertension, cardiovascular diseases and diabetic complications. Possible mechanisms of the amelioration may involve suppression of inflammation (cytokine storm), inflammasome, oxidative stress, and carbonyl stress, regulation of Ca^2+^ influx, elevation of carnosine (a cardioprotector), and immune function improvement.

Vitamin B6 is a water-soluble vitamin found in various foods such as fish, whole grains, and banana (10). There are six isoforms of B6 vitamers (10). Among these, pyridoxal 5′-phosphate (PLP) is the most active form that acts as a coenzyme in various enzymatic reactions (10). There is growing evidence that vitamin B6 exerts a protective effect against chronic diseases such as cardiovascular diseases (CVD) and diabetes by suppressing inflammation, inflammasomes, oxidative stress, and carbonyl stress (11). Additionally, vitamin B6 deficiency is associated with lower immune function and higher susceptibility to viral infection (12, 13). In view of these information, we postulated potential role of vitamin B6 in ameliorating the severity of COVID-19 and its complications (Figure 1). In this article, we review precedent research to test this hypothesis.

## Pathogenesis of COVID-19 Infection

### COVID-19 and Its Complications

Recent studies suggest that COVID-19 patients are likely to have chronic diseases (32–86%), among which hypertension (15–49%), CVD (6–40%), and diabetes (10–30%) (4–9) are most commonly reported worldwide. For example, in China, of 138 patients, 46% had a pre-existing chronic disease, including hypertension (31%), CVD (15%), and diabetes (10%) (5). In Italy, of 1,043 patients, 68% had chronic diseases, and hypertension was the most common comorbidity (49%), followed by CVD (21%) and diabetes (17%) (7). In the United States, of 7,162 cases, 38% had chronic diseases, with the top three being diabetes (10%), chronic lung disease (9.2%), and CVD (9.0%) (8). In Thailand, the first country outside of China to report a COVID-19 case (14), among the 54 patients who died (up to May 2, 2020), 56% had underlying diseases, and the top three diseases were diabetes (30%), hypertension (17%), kidney disease (13%), and CVD (6%) (Supplementary Table) (9). Besides these comorbidities, high cumulative incidence of thrombotic complications was found in critically ill patients with COVID-19 pneumonia in Dutch hospitals (15). In addition to the above data, evidence suggests that male and older populations are more susceptible to viral infection (4–8). Thus, these groups and patients with chronic diseases are more likely to require critical care.

### COVID-19 and Endothelial Cell Inflammation

COVID-19 is caused by SARS-CoV2. The virus mainly enters the body by binding to the angiotensin converting enzyme (ACE) 2 receptor, which is highly expressed in lung alveolar cells and epithelial cells in the respiratory tract, thus causing lung injury (1). Besides cells in the airways, the ACE2 receptor is highly expressed in cardiac and vascular endothelial cells (1, 2), which potentially make heart and blood vessels target organs for the virus. This may explain common cardiovascular complications with poor outcomes in COVID-19 patients with CVD and hypertension. Post-mortem analysis of COVID-19 patients revealed direct viral infection and inflammation of endothelial cells causing endothelial dysfunction and apoptosis subsequent to vascular leakage in many organs (2). Since blood vessels traverse multiple organs, this may partly explain systemic inflammation and multi-organ failure, commonly found in COVID-19 patients.

Another feature of COVID-19 is cytokine storm, resulting from excessive and aberrant host immune responses (1, 3). Studies of COVID-19 patients' serum show lymphopenia and a marked increase in inflammatory markers such as interleukin-6 (IL-6) and C-reactive protein (CRP). These are commonly observed parameters linked to disease severity (4–6). Thus, it can be hypothesized that for chronic inflammatory diseases such as diabetes where the immune response is impaired, abnormal immune activation and hyperinflammation in COVID-19 possibly make patients more susceptible to viral infection.

Taken together, agents that can moderate immune function and inflammation, maintain endothelial cell integrity, and ameliorate chronic diseases may be useful in reducing the severity of and/or curing COVID-19. In this paper, we will analyze this problem in terms of nutrition. The rationale for using vitamin B6 as a possible adjuvant treatment for COVID-19 is discussed in the following sections.

## Vitamin B6 and Cardiovascular Diseases

Evidence suggests that a low dietary intake of vitamin B6 is associated with a high risk of mortality risk from CVD, and vitamin B6 supplementation reduces this risk (11, 16). In humans, low PLP plasma levels are associated with a high risk of CVD, atherosclerosis, stroke, and thrombosis (11, 16). Recently, the role of vitamin B6 in CVD risk has been addressed through chronic inflammation, a crucial mechanism underlying atherosclerosis and CVD progression. Plasma PLP levels were inversely correlated with systemic inflammation markers such as CRP (17). Vitamin B6 supplementation suppressed IL-6 and increased total lymphocytes in patients with chronic conditions (18). Notably, both an increase in CRP and IL-6 and a decrease in lymphocytes were common in COVID-19 patient sera (4–6). Recently, novel heart protective effects of vitamin B6 have been proposed such as regulating homeostasis of imidazole dipeptides, e.g., carnosine and anserine which are cardioprotectors with antioxidant and anti-inflammatory activities (11, 19). Vitamin B6 can also regulate cellular calcium influx through both voltage-mediated and ATP-mediated purinergic mechanisms, which suggests its role in regulating hypertension and cardio-dysfunction (20). In line with this, vitamin B6 supplementation showed blood pressure lowering effect in hypertensive patients (21). Additionally, oral administration of vitamin B6 attenuates platelet aggregation and clot formation (22). Taken together, it can be suggested that vitamin B6 may ameliorate the severity of COVID-19 by preventing worsening of CVD complications through those beneficial actions.

## Vitamin B6 and Diabetes

Vitamin B6 has been found to be associated with diabetes, wherein blood PLP levels are lower in these patients (23). Studies have demonstrated that vitamin B6 supplementation reduces the incidence of diabetes and its complications (24, 25). Vitamin B6 deficiency is associated with insulin-glucagon dysregulation, glucose tolerance, and β-cell degeneration (24). Since vascular disease is a hallmark of diabetic complications, this may explain the comorbidities of CVD, hypertension, and diabetes in COVID-19. Vitamin B6 was even found to play a beneficial role in vascular endothelial function in diabetic patients (26). Among the B6-vitamers, pyridoxamine has anti-glycation activity and inhibits the formation of advanced glycation end-products (AGEs) that are major mediators of inflammation, oxidative stress, and endothelial-vascular wall damage (27). Increase in AGEs is implicated in initiation and progression of diabetes-associated microvascular diseases, major diabetic complications. Based on these notions, we can assume that sufficient vitamin B6 levels are beneficial to suppress severity of COVID-19, partly through ameliorating diabetic complications.

## Vitamin B6 and Pneumonia

In 1949, Leftwich and Mirick reported a preventive effect of vitamin B6 against viral infection (13). Mice fed a vitamin B6-deficient diet were more susceptible to infection of murine pneumonia virus than control mice. Shan et al. recently indicated that vitamin B6 administration remarkably inhibited LPS-induced systemic inflammation and acute pneumonia in mice (28). Key events linked to infection with respiratory viruses are associated with oxidative stress, inflammation, and subsequent lung injury. In fact, oral administration of anti-oxidants such as carnosine and N-acetylcysteine exerted beneficial effects on lung injury (29, 30). These suggest that vitamin B6 may ameliorate the severity of COVID-19 by exerting its anti-oxidative and anti-inflammatory actions in lung, a primary target organ for COVID-19 virus infection.

## Vitamin B6 and Immune Function

Vitamin B6 supplementation improved immune function in both human and animal studies (10), and vitamin B6 deficiency led to impairment of various facets of immunity such as lymphoid atrophy and reduced lymphocyte numbers (12). It improves the immune response, causing increased antibody production, and enhances communicative interactions between cytokines and chemokines (31). Thus, its deficiency may lead to suppressed immunity predisposing patients to infections. A previous study has implicated the lipid mediator sphingosine 1-phosphate (S1P) in vitamin B6-mediated immune regulation (32, 33). S1P regulates cell trafficking, especially cell egress from organized lymphoid tissues in thymus, bone marrow, lymph nodes, and intestinal mucosa (33). Cell trafficking is determined by the S1P gradient through S1P production and degradation mediated by S1P lyase and S1P phosphohydrolase (33). Since S1P lyase requires PLP as a coenzyme for S1P degradation, its deficiency impairs S1P lyase activity and elevates S1P levels. This in turn impairs lymphocyte trafficking from lymphoid tissues and reduces lymphocyte numbers in the peripheral tissues (32, 33).

## Vitamin B6 and Inflammasome

Canonical inflammasomes are cytoplasmic multiprotein complexes that activates caspase-1 in response to a variety of physiological and pathogenic stimuli (11, 34), leading to a downstream cascade of inflammatory response through the release of inflammatory modulators, such as Interleukin-1β (IL-1β) and interleukin-18 (IL-18) (11, 34). Among the various inflammasomes, NLRP3 inflammasome in macrophages, endothelial cells, and epithelial cells responds to a broad variety of stimuli, particularly viral RNA and its components (35, 36). NLRP3 inflammasome activation plays an important role in virus clearing by innate immunity; however, overactivation promotes inflammatory cell and host cell death (11, 34). Recently, vitamin B6 was shown to reduce IL-1β production by suppressing NLRP3 inflammasome responded to various NLRP3 inflammasome stimuli (37). Furthermore, vitamin B6 markedly reduced reactive oxygen species (ROS) production in peritoneal macrophages, where it plays a central role in NLRP3 inflammasome activation (37). It is suggested that at an early stage in the infection to macrophages, endothelial cells, and epithelial cells, SARS-CoV2 potentially escapes innate immune, thereby increasing the viral replication (1). Then, those infected cells undergo cell death, causing acute virus spread and severe cytokine storm. Here, we assume that vitamin B6 is possible to suppress hyperinflammation, at least in part, through NLRP3 inflammasome inhibition, limiting virus spread and cytokine storm. Together with the notion that NLRP3 inflammasome plays a central role in chronic diseases, including CVD, diabetes, and acute viral pneumonia (38–40), the anti-inflammasome effect of vitamin B6 suggest its therapeutic role in reducing the severity of COVID-19 and its complications.

## Vitamin B6 and Oxidative Stress

Since the discovery of B6-vitamers as ROS scavengers (41, 42), evidence indicates an inverse association between vitamin B6-deficient status and higher oxidative stress (11, 43). Studies indicate that B6-vitamers can reduce superoxide radical and lipid peroxide levels induced by H_2_O_2_ in vascular endothelial cells (44). Emerging evidence suggests that hydrogen sulfide (H_2_S) exerts strong anti-oxidant and anti-inflammatory effects at low levels (45). In the liver and cardiovascular tissues, H_2_S formation involves a PLP-dependent enzyme, cystathionine β-lyase, which is affected by vitamin B6 levels (45). Our recent studies revealed that vitamin B6 supplementation to a marginal-vitamin B6 deficient diet caused a remarkable increase in levels of imidazole dipeptides, carnosine and anserine, in heart and skeletal muscle of rats, possibly by modulating PLP-enzymes for biosynthesis (11, 19). Carnosine has various health benefits, including anti-oxidant, anti-inflammatory, anti-glycation, anti-ischemic, anti-cognitive, anti-aging, and ergogenic effects (11, 46). Thus, vitamin B6 may help to maintain a healthy defense system for the body to fight oxidative stress associated with virus infection.

## Conclusion

Here, we summarized the available evidence suggesting the potential role of vitamin B6 in suppressing the severity of COVID-19 possibly through ameliorating complications of chronic diseases such as hypertension, CVD, and diabetes. Clinical studies in COVID-19 patients are urgently needed to confirm these possibilities. In spite of the lungs being a primary target organ for SARS-CoV2 infection, information regarding the role of nutrition in lung health is very limited. Considering the emergence of new viruses, nutrition studies on the lungs, a primary target of airborne viral infections, should be performed. Severe vitamin B6 deficiency is relatively uncommon, but some individuals might have marginal vitamin B6 deficiency. Vitamin B6 can be easily available as a dietary supplement with low cost and health risk. Accumulating evidence suggests that vitamin B6 supplementation may be useful for COVID-19 patients with low vitamin B6 status.

## Author Contributions

All authors listed have made a substantial, direct and intellectual contribution to the work, and approved it for publication.

## Conflict of Interest

The authors declare that the research was conducted in the absence of any commercial or financial relationships that could be construed as a potential conflict of interest.
