# Supplementary material for: Potential Role of Vitamin B6 in Ameliorating the Severity of COVID-19 and Its Complications
Source: Front Nutr. 2020 Oct 29;7:562051. doi: 10.3389/fnut.2020.562051 (PMC7658555; doi:10.3389/fnut.2020.562051)
Supplement: Supplementary file 1 [file Table_1.docx]

Supplementary Material

# Supplementary Table Underlying disease information of 54 patients who died from COVID-19 in Thailand (up to May 2, 2020)

| **Average age** (Years) | | 59 |  |
| --- | --- | --- | --- |
| **Male** (%) | | 76 |  |
| **Female** (%) | | 24 |  |
| **Underlying diseases/conditions** (%) | | 55.6 |  |
| Diabetes (%) | | 29.6 |  |
| Hypertension (%) | | 16.7 |  |
| Dyslipidemia (%) | | 14.8 |  |
| Kidney disease (%) | | 13.0 |  |
| Obesity (%) | | 13.0 |  |
| Heart disease (%) | | 5.6 |  |
| Emphysema (%) | | 1.9 |  |
| Gout (%) | | 1.9 |  |
| Anemia (%) | | 1.9 |  |
| Cerebrovascular disease (%) | | 1.9 |  |
| Tuberculosis (%) | | 1.9 |  |
| Liver disease (%) | | 1.9 |  |
| Systemic Lupus Erythematosus (%) | | 1.9 |  |
| Cancer (%) | | 1.9 |  |
| No underlying diseases (%) | | 5.6 |  |
| Not reported (%) | | 38.9 |  |
| **Gender** | **Age (years)** | **Nationality** | **Underlying diseases/conditions** |
| Male | 35 | Thai | Not reported |
| Male | 70 | Thai | Tuberculosis |
| Male | 79 | Thai | Various underlying diseases, but not specified |
| Male | 45 | Thai | Diabetes/Obesity |
| Male | 50 | Thai | Not reported |
| Female | 55 | Thai | Diabetes/Dyslipidemia |
| Male | 68 | Thai | Not reported |
| Male | 54 | Thai | Not reported |
| Female | 56 | Thai | Not reported |
| Male | 48 | Thai | Diabetes/Cancer/Kidney disease/liver disease |
| Male | 79 | Thai | Diabetes/ Kidney disease |
| Male | 58 | Thai | Not reported |
| Male | 57 | Thai | Not reported |
| Male | 77 | Thai | Pulmonary Emphysema/Diabetes |
| Male | 55 | Thai | Not reported |
| Male | 59 | Thai | Not reported |
| Male | 72 | Thai | Kidney disease |
| Male | 84 | Thai | Kidney disease/Hypertension/Gout |
| Male | 84 | Thai | Not reported |
| Male | 72 | Not clear | Hypertension/Diabetes/Dyslipidemia |
| Male | 46 | Thai | Not reported |
| Male | 82 | Swiss | Heart disease/Hypertension/Dyslipidemia |
| Male | 30 | Thai | Not reported, but alcoholism |
| Male | 28 | Thai | Not reported |
| Male | 51 | Thai | Diabetes/Hypertension/Obesity |
| Female | 59 | Thai | Diabetes |
| Male | 54 | Thai | No underlying diseases |
| Male | 48 | Russian | Not reported |
| Male | 69 | Indian | Diabetes/ Heart disease |
| Male | 69 | American | Kidney disease |
| Male | 74 | French | No underlying diseases |
| Male | 82 | Thai | Not reported |
| Female | 43 | Thai | Systemic Lupus Erythematosus |
| Male | 46 | Thai | Obesity |
| Male | 65 | Thai | Not reported |
| Male | 74 | Thai | Diabetes/Dyslipidemia |
| Female | 65 | Thai | Obesity/Dyslipidemia |
| Male | 44 | Thai | Not reported |
| Male | 56 | Thai | Not reported |
| Male | 43 | Thai | Diabetes/Kidney disease/Dyslipidemia |
| Female | 52 | Thai | Hypertension/Heart disease |
| Female | 65 | Thai | Diabetes/Kidney disease/Hypertension |
| Not identified | 60 | Thai | Not reported |
| Male | 55 | Malaysian | No underlying diseases |
| Female | 35 | Thai | Diabetes/Dyslipidemia |
| Male | 37 | Thai | Hypertension/Obesity |
| Female | 85 | Thai | Diabetes/Hypertension |
| Male | 50 | Thai | Diabetes/Smoking |
| Female | 58 | Thai | Diabetes/Hypertension/Dyslipidemia/Obesity |
| Female | 78 | Thai | Cerebrovascular disease |
| Male | 48 | Thai | Not reported |
| Female | 64 | Thai | Anemia |
| Male | 52 | Thai | Not reported |
| Female | 63 | Thai | Obesity |

**Note:** The data have been extracted from the Thai government's announcement regarding COVID-19 situation, which was daily announced by Thai government spokesman Major General Werachon Sukondhapatipak and broadcasted by Thai PBS channel. In the announcements, the general information of the dead patients, including age, gender, nationality, and underlying diseases, was announced publicly. The data shown here are publicly accessible. We, the authors, do not own the data.

Since dyslipidemia is rather a chronic condition than a chronic disease, we excluded it from the list of chronic diseases. Thus, the top three chronic diseases are diabetes, hypertension, and kidney disease.
